# 1-(2-Chloro­benzo­yl)-3-[4-(trifluoro­meth­oxy)phen­yl]urea

**DOI:** 10.1107/S1600536808016462

**Published:** 2008-06-07

**Authors:** Yin-hong Liu, Fang-shi Li, Li-he Yin, Da-sheng Yu

**Affiliations:** a Department of Applied Chemistry, College of Science, Nanjing University of Technology, Xinmofan Road No. 5, Nanjing 210009, People’s Republic of China

## Abstract

The title compound, C_15_H_10_ClF_3_N_2_O_3_, is considered to belong to a fourth generation of insecticides with properties such as high selectivity, low acute toxicity for mammals and high biological activity. The dihedral angle between the two benzene rings is 59.3 (2)°. Intra­molecular C—H⋯O and N—H⋯O hydrogen bonds are observed. Inter­molecular N—H⋯O hydrogen bonding generates a centrosymmetric dimer. The F atoms are disordered over two positions; the site occupancy factors are 0.52 and 0.48.

## Related literature

For related literature, see: Allen *et al.* (1987 ▶); Wang *et al.* (1998 ▶); Qiu *et al.* (12004 ▶).
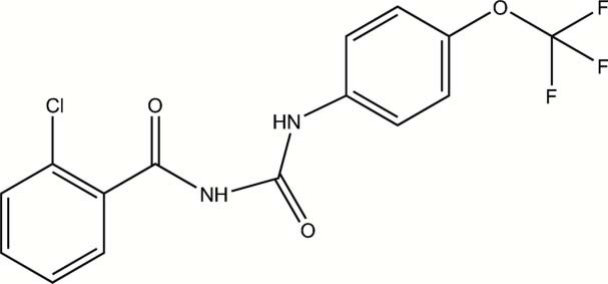


## Experimental

### 

#### Crystal data

C_15_H_10_ClF_3_N_2_O_3_
*M* *_r_* = 358.70
Monoclinic, 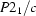

*a* = 17.293 (4) Å
*b* = 8.2870 (17) Å
*c* = 11.073 (2) Å
β = 101.74 (3)°
*V* = 1553.6 (6) Å^3^
*Z* = 4
Mo *K*α radiation
μ = 0.30 mm^−1^
*T* = 298 (2) K
0.30 × 0.20 × 0.20 mm

#### Data collection

Enraf–Nonius CAD-4 diffractometer
Absorption correction: ψ scan (North *et al.*, 1968 ▶) *T*_min_ = 0.917, *T*_max_ = 0.943
2946 measured reflections
2784 independent reflections
1906 reflections with *I* > 2σ(*I*)
*R*_int_ = 0.012
3 standard reflections every 200 reflections intensity decay: none

#### Refinement

*R*[*F* ^2^ > 2σ(*F* ^2^)] = 0.079
*wR*(*F* ^2^) = 0.186
*S* = 1.01
2784 reflections
209 parameters
1 restraint
H-atom parameters constrained
Δρ_max_ = 0.58 e Å^−3^
Δρ_min_ = −0.38 e Å^−3^

### 

Data collection: *CAD-4 Software* (Enraf–Nonius, 1989 ▶); cell refinement: *CAD-4 Software*; data reduction: *XCAD4* (Harms & Wocadlo, 1995 ▶); program(s) used to solve structure: *SHELXS97* (Sheldrick, 2008 ▶); program(s) used to refine structure: *SHELXL97* (Sheldrick, 2008 ▶); molecular graphics: *SHELXTL* (Sheldrick, 2008 ▶); software used to prepare material for publication: *SHELXTL*.

## Supplementary Material

Crystal structure: contains datablocks global, I. DOI: 10.1107/S1600536808016462/kp2174sup1.cif

Structure factors: contains datablocks I. DOI: 10.1107/S1600536808016462/kp2174Isup2.hkl

Additional supplementary materials:  crystallographic information; 3D view; checkCIF report

## Figures and Tables

**Table 1:** Hydrogen-bond geometry (Å, °)

*D*—H⋯*A*	*D*—H	H⋯*A*	*D*⋯*A*	*D*—H⋯*A*
N1—H1*A*⋯O3	0.86	1.95	2.653 (4)	138
N2—H2*A*⋯O2^i^	0.86	2.00	2.851 (4)	172
C6—H6*A*⋯O2	0.93	2.24	2.838 (5)	121

Symmetry code: (i) 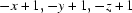.

**Fig. 1.**
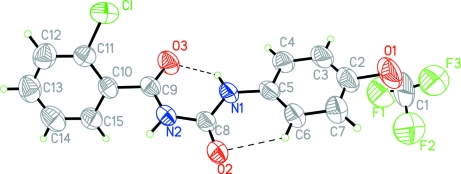
The molecular structure of (I) with the atom-numbering scheme. Displacement ellipsoids are drawn at the 50% probability level. Intramolecular hydrogen bonds are shown by dased lines.

**Fig. 2.**
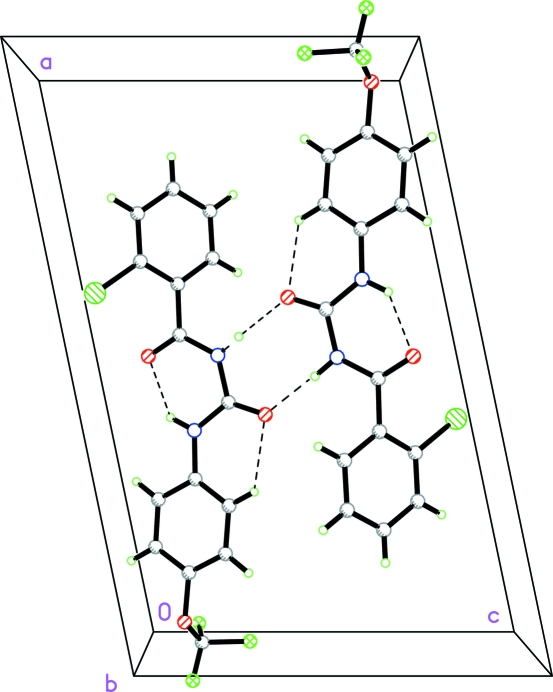
The crystal packing diagram with hydrogen bonds drawn as dashed lines.

## supplementary crystallographic information

### Comment

The title compound, (I), is generally recognized as an insect growth regulator that interferes with chitin synthesis in target pests causing death or abortive development (Wang *et al.*,  1998). As part of our studies in this area, we report herein the crystal structure of the title compound (I).

In the molecule of (I) (Fig.1), the bond lengths and angles are within normal ranges (Allen *et al.*, 1987). The intramolecular C—H···O and N—H···O hydrogen bonds are observed (Fig. 1, Table 1). Intermolecular N—H···O hydrogen bond generates a cyclic, centrosymmetric hydrogen bonded dimer (Table 1, Fig. 2).

### Experimental

The title compound, (I), was prepared according to the literature method (Qiu *et al.*, 1981). The crystals suitable for X-ray analysis were obtained by dissolving (I) (0.1 g) in acetonitrile (25 mL) and evaporating the solvent slowly at room temperature for about 6 d.

### Refinement

H atoms were positioned geometrically, C—H = 0.93 Å for aromatic, N—H = 0.86 Å for amido H, and constrained to ride on their parent atoms, with *U*_iso_(H) = *xU*_eq_(C,*N*), where *x* = 1.2 for all the H atoms.

Trifloromethyl group was disordered over two sites, occupancies were refined and converged to 0.52 and 0.48, respectively.

### Crystal data

**Table:**

C_15_H_10_ClF_3_N_2_O_3_	*F*_000_ = 728
*M**_r_* = 358.70	*D*_x_ = 1.534 Mg m^−^^3^
Monoclinic, *P*2_1_/*c*	Mo *K*α radiation λ = 0.71073 Å
Hall symbol: -P 2ybc	Cell parameters from 25 reflections
*a* = 17.293 (4) Å	θ = 10–14º
*b* = 8.2870 (17) Å	µ = 0.30 mm^−^^1^
*c* = 11.073 (2) Å	*T* = 298 (2) K
β = 101.74 (3)º	Block, colourless
*V* = 1553.6 (6) Å^3^	0.30 × 0.20 × 0.20 mm
*Z* = 4	

### Data collection

**Table:**

Enraf–Nonius CAD-4 diffractometer	*R*_int_ = 0.012
Radiation source: fine-focus sealed tube	θ_max_ = 25.2º
Monochromator: graphite	θ_min_ = 1.2º
*T* = 298(2) K	*h* = −20→20
ω/2θ scans	*k* = 0→9
Absorption correction: ψ scan(North *et al.*, 1968)	*l* = 0→13
*T*_min_ = 0.917, *T*_max_ = 0.943	3 standard reflections
2946 measured reflections	every 200 reflections
2784 independent reflections	intensity decay: none
1906 reflections with *I* > 2σ(*I*)	

### Refinement

**Table:**

Refinement on *F*^2^	Secondary atom site location: difference Fourier map
Least-squares matrix: full	Hydrogen site location: inferred from neighbouring sites
*R*[*F*^2^ > 2σ(*F*^2^)] = 0.079	H-atom parameters constrained
*wR*(*F*^2^) = 0.186	*w* = 1/[σ^2^(*F*_o_^2^) + (0.06*P*)^2^ + 3*P*] where *P* = (*F*_o_^2^ + 2*F*_c_^2^)/3
*S* = 1.01	(Δ/σ)_max_ < 0.001
2784 reflections	Δρ_max_ = 0.58 e Å^−^^3^
209 parameters	Δρ_min_ = −0.37 e Å^−^^3^
1 restraint	Extinction correction: none
Primary atom site location: structure-invariant direct methods	

### Special details

**Table:**

Geometry. All e.s.d.'s (except the e.s.d. in the dihedral angle between two l.s. planes) are estimated using the full covariance matrix. The cell e.s.d.'s are taken into account individually in the estimation of e.s.d.'s in distances, angles and torsion angles; correlations between e.s.d.'s in cell parameters are only used when they are defined by crystal symmetry. An approximate (isotropic) treatment of cell e.s.d.'s is used for estimating e.s.d.'s involving l.s. planes.
Refinement. Refinement of *F*^2^ against ALL reflections. The weighted *R*-factor *wR* and goodness of fit *S* are based on *F*^2^, conventional *R*-factors *R* are based on *F*, with *F* set to zero for negative *F*^2^. The threshold expression of *F*^2^ > σ(*F*^2^) is used only for calculating *R*-factors(gt) *etc*. and is not relevant to the choice of reflections for refinement. *R*-factors based on *F*^2^ are statistically about twice as large as those based on *F*, and *R*- factors based on ALL data will be even larger.

### Fractional atomic coordinates and isotropic or equivalent isotropic displacement parameters (Å^2^)

**Table:**

	*x*	*y*	*z*	*U*_iso_*/*U*_eq_	Occ. (<1)
Cl	0.39377 (8)	0.46678 (16)	0.93229 (10)	0.0767 (4)	
F1	0.9765 (5)	0.8806 (12)	0.8631 (10)	0.147	0.52
F2	0.9962 (5)	0.7066 (11)	0.7283 (8)	0.131	0.52
F3	1.0673 (5)	0.7391 (10)	0.9039 (8)	0.127	0.52
F1'	0.9962 (6)	0.8363 (13)	0.9452 (9)	0.145	0.48
F2'	0.9788 (6)	0.7985 (12)	0.7564 (10)	0.140	0.48
F3'	1.0763 (4)	0.6519 (9)	0.8854 (7)	0.111	0.48
O1	0.9560 (2)	0.6074 (6)	0.8858 (4)	0.1185 (16)	
O2	0.59902 (15)	0.5358 (4)	0.5609 (2)	0.0661 (8)	
O3	0.50002 (17)	0.7159 (4)	0.8415 (3)	0.0733 (9)	
N1	0.62777 (18)	0.6359 (4)	0.7569 (3)	0.0578 (9)	
H1A	0.6061	0.6732	0.8145	0.069*	
N2	0.49834 (18)	0.6012 (4)	0.6534 (3)	0.0552 (8)	
H2A	0.4665	0.5691	0.5876	0.066*	
C1	0.9996 (3)	0.7289 (13)	0.8574 (9)	0.146 (3)	
C2	0.8720 (3)	0.6213 (7)	0.8485 (5)	0.0820 (15)	
C3	0.8311 (3)	0.6917 (7)	0.9265 (5)	0.0849 (16)	
H3A	0.8573	0.7353	1.0010	0.102*	
C4	0.7488 (3)	0.6978 (6)	0.8930 (4)	0.0742 (13)	
H4A	0.7194	0.7466	0.9445	0.089*	
C5	0.7113 (2)	0.6298 (5)	0.7812 (3)	0.0570 (10)	
C6	0.7545 (2)	0.5566 (6)	0.7047 (4)	0.0694 (12)	
H6A	0.7294	0.5092	0.6310	0.083*	
C7	0.8363 (3)	0.5553 (7)	0.7400 (5)	0.0802 (14)	
H7A	0.8666	0.5088	0.6888	0.096*	
C8	0.5786 (2)	0.5883 (5)	0.6510 (4)	0.0532 (10)	
C9	0.4632 (2)	0.6571 (5)	0.7445 (4)	0.0527 (9)	
C10	0.3755 (2)	0.6418 (5)	0.7208 (4)	0.0539 (10)	
C11	0.3384 (2)	0.5638 (5)	0.8036 (4)	0.0561 (10)	
C12	0.2570 (3)	0.5556 (6)	0.7844 (5)	0.0755 (13)	
H12A	0.2327	0.5031	0.8410	0.091*	
C13	0.2120 (3)	0.6263 (7)	0.6803 (5)	0.0842 (15)	
H13A	0.1572	0.6227	0.6672	0.101*	
C14	0.2478 (3)	0.7012 (7)	0.5968 (5)	0.0842 (15)	
H14A	0.2172	0.7477	0.5267	0.101*	
C15	0.3293 (3)	0.7087 (6)	0.6157 (4)	0.0687 (12)	
H15A	0.3532	0.7588	0.5576	0.082*	

### Atomic displacement parameters (Å^2^)

**Table:**

	*U*^11^	*U*^22^	*U*^33^	*U*^12^	*U*^13^	*U*^23^
Cl	0.0925 (9)	0.0813 (8)	0.0561 (6)	0.0109 (7)	0.0145 (6)	0.0084 (6)
F1	0.147	0.147	0.147	0.000	0.030	0.000
F2	0.131	0.131	0.131	0.000	0.027	0.000
F3	0.127	0.127	0.127	0.000	0.026	0.000
F1'	0.145	0.145	0.145	0.000	0.030	0.000
F2'	0.140	0.140	0.140	0.000	0.029	0.000
F3'	0.111	0.111	0.111	0.000	0.023	0.000
O1	0.0479 (19)	0.170 (4)	0.123 (3)	−0.013 (2)	−0.017 (2)	0.038 (3)
O2	0.0487 (15)	0.092 (2)	0.0543 (16)	−0.0083 (15)	0.0024 (13)	−0.0147 (16)
O3	0.0656 (18)	0.083 (2)	0.0648 (18)	−0.0052 (16)	−0.0016 (15)	−0.0225 (17)
N1	0.0467 (18)	0.072 (2)	0.0491 (18)	−0.0096 (16)	−0.0039 (14)	−0.0009 (17)
N2	0.0502 (18)	0.064 (2)	0.0468 (17)	−0.0067 (16)	−0.0008 (14)	−0.0074 (16)
C1	0.025 (2)	0.234 (10)	0.168 (7)	−0.022 (4)	−0.005 (3)	0.020 (8)
C2	0.050 (3)	0.106 (4)	0.080 (3)	−0.017 (3)	−0.010 (2)	0.024 (3)
C3	0.056 (3)	0.126 (5)	0.063 (3)	−0.028 (3)	−0.012 (2)	0.007 (3)
C4	0.062 (3)	0.097 (4)	0.059 (3)	−0.023 (3)	−0.002 (2)	−0.001 (2)
C5	0.052 (2)	0.066 (3)	0.048 (2)	−0.014 (2)	−0.0026 (18)	0.0114 (19)
C6	0.049 (2)	0.089 (3)	0.063 (3)	−0.003 (2)	−0.004 (2)	0.002 (2)
C7	0.055 (3)	0.098 (4)	0.084 (3)	−0.001 (3)	0.006 (2)	0.004 (3)
C8	0.049 (2)	0.059 (2)	0.047 (2)	−0.0097 (19)	0.0009 (17)	−0.0001 (19)
C9	0.056 (2)	0.046 (2)	0.052 (2)	−0.0018 (18)	0.0009 (18)	0.0000 (18)
C10	0.053 (2)	0.047 (2)	0.058 (2)	0.0019 (18)	0.0017 (18)	−0.0064 (19)
C11	0.063 (2)	0.055 (3)	0.051 (2)	0.0033 (19)	0.0140 (19)	−0.0095 (19)
C12	0.066 (3)	0.082 (3)	0.083 (3)	0.002 (3)	0.027 (3)	−0.007 (3)
C13	0.057 (3)	0.098 (4)	0.097 (4)	0.007 (3)	0.013 (3)	−0.011 (3)
C14	0.071 (3)	0.088 (4)	0.082 (3)	0.022 (3)	−0.011 (3)	0.002 (3)
C15	0.061 (3)	0.077 (3)	0.064 (3)	0.005 (2)	0.003 (2)	0.012 (2)

### Geometric parameters (Å, °)

**Table:**

Cl—C11	1.743 (4)	C3—C4	1.397 (6)
F1—C1	1.324 (12)	C3—H3A	0.9300
F2—C1	1.431 (11)	C4—C5	1.394 (6)
F3—C1	1.181 (10)	C4—H4A	0.9300
F1'—C1	1.327 (12)	C5—C6	1.379 (6)
F2'—C1	1.244 (11)	C6—C7	1.389 (6)
F3'—C1	1.447 (10)	C6—H6A	0.9300
O1—C1	1.334 (9)	C7—H7A	0.9300
O1—C2	1.432 (5)	C9—C10	1.490 (5)
O2—C8	1.205 (4)	C10—C11	1.382 (6)
O3—C9	1.233 (4)	C10—C15	1.386 (5)
N1—C8	1.360 (5)	C11—C12	1.381 (6)
N1—C5	1.416 (5)	C12—C13	1.383 (7)
N1—H1A	0.8600	C12—H12A	0.9300
N2—C9	1.361 (5)	C13—C14	1.362 (7)
N2—C8	1.398 (5)	C13—H13A	0.9300
N2—H2A	0.8600	C14—C15	1.384 (6)
C2—C7	1.350 (7)	C14—H14A	0.9300
C2—C3	1.354 (7)	C15—H15A	0.9300
			
C1—O1—C2	117.4 (5)	C5—C4—H4A	120.5
C8—N1—C5	126.0 (4)	C3—C4—H4A	120.5
C8—N1—H1A	117.0	C6—C5—C4	120.8 (4)
C5—N1—H1A	117.0	C6—C5—N1	123.9 (4)
C9—N2—C8	129.5 (3)	C4—C5—N1	115.2 (4)
C9—N2—H2A	115.3	C5—C6—C7	118.6 (4)
C8—N2—H2A	115.3	C5—C6—H6A	120.7
F3—C1—F2'	115.9 (9)	C7—C6—H6A	120.7
F3—C1—F1	101.2 (9)	C2—C7—C6	120.2 (5)
F2'—C1—F1	64.4 (8)	C2—C7—H7A	119.9
F3—C1—F1'	79.7 (8)	C6—C7—H7A	119.9
F2'—C1—F1'	107.4 (11)	O2—C8—N1	125.6 (4)
F1—C1—F1'	43.2 (6)	O2—C8—N2	120.2 (3)
F3—C1—O1	120.6 (9)	N1—C8—N2	114.2 (4)
F2'—C1—O1	119.2 (7)	O3—C9—N2	123.4 (4)
F1—C1—O1	121.0 (7)	O3—C9—C10	120.9 (4)
F1'—C1—O1	102.8 (8)	N2—C9—C10	115.7 (3)
F3—C1—F2	106.3 (8)	C11—C10—C15	118.6 (4)
F2'—C1—F2	38.4 (6)	C11—C10—C9	121.1 (3)
F1—C1—F2	102.6 (9)	C15—C10—C9	120.4 (4)
F1'—C1—F2	145.0 (11)	C12—C11—C10	121.1 (4)
O1—C1—F2	103.1 (8)	C12—C11—Cl	118.5 (4)
F3—C1—F3'	32.4 (5)	C10—C11—Cl	120.4 (3)
F2'—C1—F3'	118.7 (9)	C11—C12—C13	119.4 (5)
F1—C1—F3'	132.9 (8)	C11—C12—H12A	120.3
F1'—C1—F3'	108.3 (8)	C13—C12—H12A	120.3
O1—C1—F3'	98.9 (8)	C14—C13—C12	120.2 (5)
F2—C1—F3'	90.4 (7)	C14—C13—H13A	119.9
C7—C2—C3	122.6 (4)	C12—C13—H13A	119.9
C7—C2—O1	118.5 (5)	C13—C14—C15	120.5 (5)
C3—C2—O1	118.8 (5)	C13—C14—H14A	119.7
C2—C3—C4	118.8 (4)	C15—C14—H14A	119.7
C2—C3—H3A	120.6	C14—C15—C10	120.3 (5)
C4—C3—H3A	120.6	C14—C15—H15A	119.9
C5—C4—C3	119.0 (5)	C10—C15—H15A	119.9

### Hydrogen-bond geometry (Å, °)

**Table:**

*D*—H···*A*	*D*—H	H···*A*	*D*···*A*	*D*—H···*A*
N1—H1A···O3	0.86	1.95	2.653 (4)	138
N2—H2A···O2^i^	0.86	2.00	2.851 (4)	172
C6—H6A···O2	0.93	2.24	2.838 (5)	121

Symmetry codes: (i) −*x*+1, −*y*+1, −*z*+1.

## References

[bb1] Allen, F. H., Kennard, O., Watson, D. G., Brammer, L., Orpen, A. G. & Taylor, R. (1987). *J. Chem. Soc. Perkin Trans. 2*, pp. S1–19.

[bb2] Enraf–Nonius (1989). *CAD-4 Software* Enraf–Nonius, Delft, The Netherlands.

[bb3] Harms, K. & Wocadlo, S. (1995). *XCAD4* University of Marburg, Germany.

[bb4] North, A. C. T., Phillips, D. C. & Mathews, F. S. (1968). *Acta Cryst.* A**24**, 351–359.

[bb6] Qiu, S. S., Li, X. Z., Zhang, S. H. & Gao, X. F. (2004). *J. Xiandai Nongyao* **3**, 17–18.

[bb7] Sheldrick, G. M. (2008). *Acta Cryst.* A**64**, 112–122. 10.1107/S0108767307043930 18156677

[bb8] Wang, S., Allan, R. D., Skerritt, J. H. & Kennedy, I. R. (1998). *J. Agric. Food Chem.* **46**, 3330–3338.

